# Identifying the High-Risk Fetus in the Low-Risk Mother Using Fetal Doppler Screening

**DOI:** 10.9745/GHSP-D-21-00692

**Published:** 2022-06-29

**Authors:** Ute Feucht, Tsakane Hlongwane, Valerie Vannevel, Helen Mulol, Tanita Botha, Robert Pattinson

**Affiliations:** a University of Pretoria, Pretoria, South Africa.; b South African Medical Research Council, Pretoria, South Africa.

## Abstract

Identifying the high-risk fetus in the low-risk pregnant mother (LRM) is a neglected area of research. Fetal growth restriction (FGR) is a major cause of stillbirths, especially in low- and middle-income countries (LMICs). FGR is very poorly detected particularly in healthy pregnant women classified as low risk. Umbiflow is an inexpensive continuous-wave Doppler ultrasound (CWDU) apparatus that is suitable for use by low-level health care providers for screening low-risk pregnant populations. It can easily detect umbilical artery blood flow in the cord, which correlates well with placental function, and poor placental function correlates well with FGR.

Use of CWDU to screen an LMIC population of more than 7,000 LRMs has demonstrated a high prevalence of abnormal umbilical artery flow of 13%, and absent end-diastolic flow, which is associated with end-stage placental disease, was found in 1.2%. This is 10 times higher than previously reported in high-income countries. Screening with CWDU together with a standard protocol managing those pregnancies with abnormal placental blood flow resulted in a 43% reduction in stillbirths (risk ratio: 0.57; 95% confidence interval=0.29, 0.85) in this LRM population. Further, follow-up of infants who had abnormal umbilical artery blood flow showed that these infants had significantly less fat-free mass at ages 6 weeks, 10 weeks, 14 weeks, and 6 months, than those with normal umbilical artery blood flow (P<.015), confirming that CWDU was able to detect true FGR.

Thus, screening with CWDU can detect the fetus at risk of stillbirth, and infants likely to have suboptimal growth and development postnatally. Screening with CWDU in LRMs opens the door to a step change in preventing stillbirths in LMICs.

## Abstract (precis)

Continuous-wave Doppler ultrasound of the umbilical artery offers an inexpensive and scalable method of detecting undiagnosed fetal growth restriction. Using Doppler to screen low-risk pregnancies in low- and middle-income countries identifies fetuses at risk of stillbirth and, when managed appropriately, results in a step change reduction in the stillbirth rate.

## Teaser

**Key Findings:**

- Infants who had an abnormal umbilical artery resistance index during pregnancy had a significantly lower fat-free mass (reflecting bone and muscle mass) at 6 weeks, 10 weeks, 14 weeks, and 6 months postpartum when compared to infants with normal umbilical artery resistance indices.
- The use of continuous-wave Doppler ultrasound can identify the fetus at risk of fetal growth restriction and allows for appropriate interventions to save the fetus and change the antenatal and postnatal trajectory.

**Key Implications:**

- Screening with continuous-wave Doppler ultrasound has the potential to save lives but may burden already strained low-resource settings.
- The identification of this group of vulnerable fetuses with abnormal Doppler results allows for further research and interventions to optimize growth and other health outcomes, both antenatally and postnatally.

## INTRODUCTION

A healthy pregnant woman expects a normal healthy baby. This happens most of the time, but occasionally it does not, and stillbirth may result. The apparent rarity of the event, and the guilt feelings of the mother and attending health care personnel often lead to this event being hidden and/or neglected.^1^^,^^2^ This causes the mother great anguish.^3^ Stillbirths occur worldwide, approximately 2 million annually, but most frequently in low- and middle-income countries (LMICs), with 3 in 4 stillbirths in sub-Saharan Africa and Southern Asia.^2^^,^^4^ It is a tragic event, the magnitude of which is grossly underestimated.^5^^,^^6^ Few interventions exist to effectively reduce stillbirths and only slow progress has been made in reducing stillbirth rates since 2000, especially when compared with neonatal and under-5 mortality.^4^^,^^7^

It is reported in demographic health surveys that there are more neonatal deaths than stillbirths, but that might be due to under-reporting, as explained by Akuze et al.^8^ In perinatal death audits, there were many more stillbirths than neonatal deaths in the United Kingdom, where there were 2,840 and 2,579 stillbirths in 2017 and 2018 respectively, while there were 1,267 and 1,199 neonatal deaths in the same 2 years.^2^^,^^9^ In South Africa, between 2017 and 2019, there were on average 14,370 stillbirths and 7,032 neonatal deaths per year.^10^ Of these stillbirths, on average, 3,838 were unexplained stillbirths (all antenatal) compared to 837 intrapartum stillbirths per year.^10^ Throughout the world, many classification systems for stillbirths are used, causing confusion; however, a striking common feature among them is that unexplained stillbirths were the most commonly reported.^11^ Most of these women would have been regarded as healthy; otherwise, the maternal condition would have been reported as the cause of the stillbirth. This stresses the point of the lack of detection of the high-risk fetus in the low-risk mother. The glossing over of this tragedy is clearly illustrated by the Sustainable Development Goals' failure to require reporting on stillbirths or set any stillbirth reduction targets.^7^

One reason why stillbirths seem to be neglected is the assumption that the remedial actions for prevention are readily available and simply require effective implementation by the health systems.^12^ Although this is true for the majority of intrapartum stillbirths, the same does not hold true for antenatal stillbirths. Most antenatal stillbirths are preterm and unexplained, and most occur in women regarded as healthy and having low-risk pregnancies.^10^^,^^13^ Even though high-risk pregnant women have a higher per-person risk of stillbirth, because most pregnant women are healthy, the majority of stillbirths in absolute numbers occur in low-risk mothers (LRMs). In these women, the most common cause of stillbirth is an undiagnosed growth-restricted fetus.^13^^–^^16^

Most antenatal care in LMICs is performed at primary health care clinics that have limited resources and a severely limited ability to detect fetal growth restriction (FGR), which is the cause of approximately half of antenatal stillbirths (T. Hlongwane, unpublished data, 2022).^17^ Routinely used techniques aimed at detecting FGR have not been shown to be effective nor prevent stillbirths, including clinical detection by symphysis-fundal height measurement and fetal movement counting.^18^^,^^19^ Even a 2-step imaging ultrasound in LMIC settings failed to show any reduction in perinatal mortality or morbidity.^20^ Therefore, antenatal stillbirths occur, especially in LMICs, despite the best antenatal care being available.

Thus, in LMICs there is currently no simple solution to prevent antenatal stillbirths. To prevent stillbirths, there needs to be an effective screening system that is accessible to all LRMs and that can detect the fetus who is likely to be growth restricted. The screening system should be simple to implement in terms of training and necessary skills, be inexpensive, require minimal human resources, and be effective in detecting at-risk fetuses. It also needs to be appropriate for introduction at the primary health care clinics where the majority of LRMs attend antenatal care.

We present a screening system using continuous-wave Doppler ultrasound (CWDU) for the fetus at risk of antenatal stillbirth. We demonstrate that, when implemented, there was a step-change reduction in the number of antenatal stillbirths and that the system is suitable for population screening in LMICs and perhaps in high-income countries (HICs).

## THE SCREENING SYSTEM

The screening system uses the CWDU technology developed in the 1980s to detect fetuses at risk of stillbirth. CWDU of the umbilical artery measures the arterial blood velocity. When blood flow is reduced due to increased resistance downstream in the placenta, this correlates very well with placental insufficiency, which is one of the major causes of FGR. CWDU detects this reduced blood flow and measures it as the resistance index (RI): the higher the RI, the more compromised the blood flow. When the placenta is damaged to the extent that no blood flow can be detected during diastole of the fetal heart, the placenta is close to failure, and the fetus is at high risk of intrauterine demise. This phenomenon is called absent end-diastolic flow (AEDF). Seven of 9 fetuses whose umbilical artery blood flow was assessed as AEDF at or beyond 28 weeks' gestation, in mothers who declined treatment, died in utero.^21^ Furthermore, acting on the result of the CWDU of the umbilical artery has been shown to reduce perinatal deaths by 29% in high-risk pregnancies.^22^ Thus, screening for AEDF, or a clearly defined abnormally high RI, could potentially reduce stillbirths if the prevalence of AEDF and abnormal RI was high enough in a pregnant population to warrant screening.

Using continuous wave Doppler ultrasound to detect and measure the resistance index has been shown to reduce perinatal deaths by 29% in high-risk pregnancies.

The Council for Scientific and Industrial Research and the South African Medical Research Council developed a low-cost CWDU device called Umbiflow. The device measures the RI in the umbilical artery (UmA-RI) and plots it against the estimated gestational age to identify fetuses at risk of FGR.^23^ CWDU detects all movements in the line of the ultrasound wave. The umbilical cord is easily detected as it is surrounded by a window of amniotic fluid and has a classic signature of the pulsatile arterial pattern flowing in one direction and a non-pulsatile wave (umbilical vein) flowing in the opposite direction. This gives the classic fingerprint which allows measurement without requiring imaging ultrasound, making it less costly than conventional imaging ultrasound. Umbiflow is a mobile-connected Doppler device consisting of a handheld proprietary Doppler probe (transducer) with a universal serial bus (USB) cable or Bluetooth, which connects to any Windows-based computer or Android-operated tablet with the necessary software. The accuracy of the Umbiflow system in measuring the UmA-RI has previously been demonstrated to be comparable to the commercial pulsed-wave Doppler units.^24^^,^^25^ Nkosi et al. reconfirmed this accuracy in a study of pregnant women who had a normal UmA-RI on CWDU examination to determine the false negative rate.^21^ Of the 226 pregnant women, only 3 had an abnormal UmA-RI when tested using the conventional ultrasound and pulsed-wave Doppler giving a false negative rate of 1.3% in this group. In the full study of 2,868 women, 355 women were referred with an abnormal UmA-RI on CWDU and 32 fetuses were found to have normal UmA-RIs when measured with the pulsed Doppler, giving a positive predictive rate of 91.0%. The advantage of CWDU is that it is considerably less expensive than pulsed-wave Doppler ultrasound, which must be performed in conjunction with imaging ultrasound. The current estimated cost of an Umbiflow device with a laptop is US$1,350, but this cost could be lowered according to the scale of implementation, together with future use of the tablet-compatible version. A portable imaging ultrasound plus pulsed-wave Doppler will cost at least US$6,400, making the CWDU apparatus at least 5 times less expensive.

**Figure:**
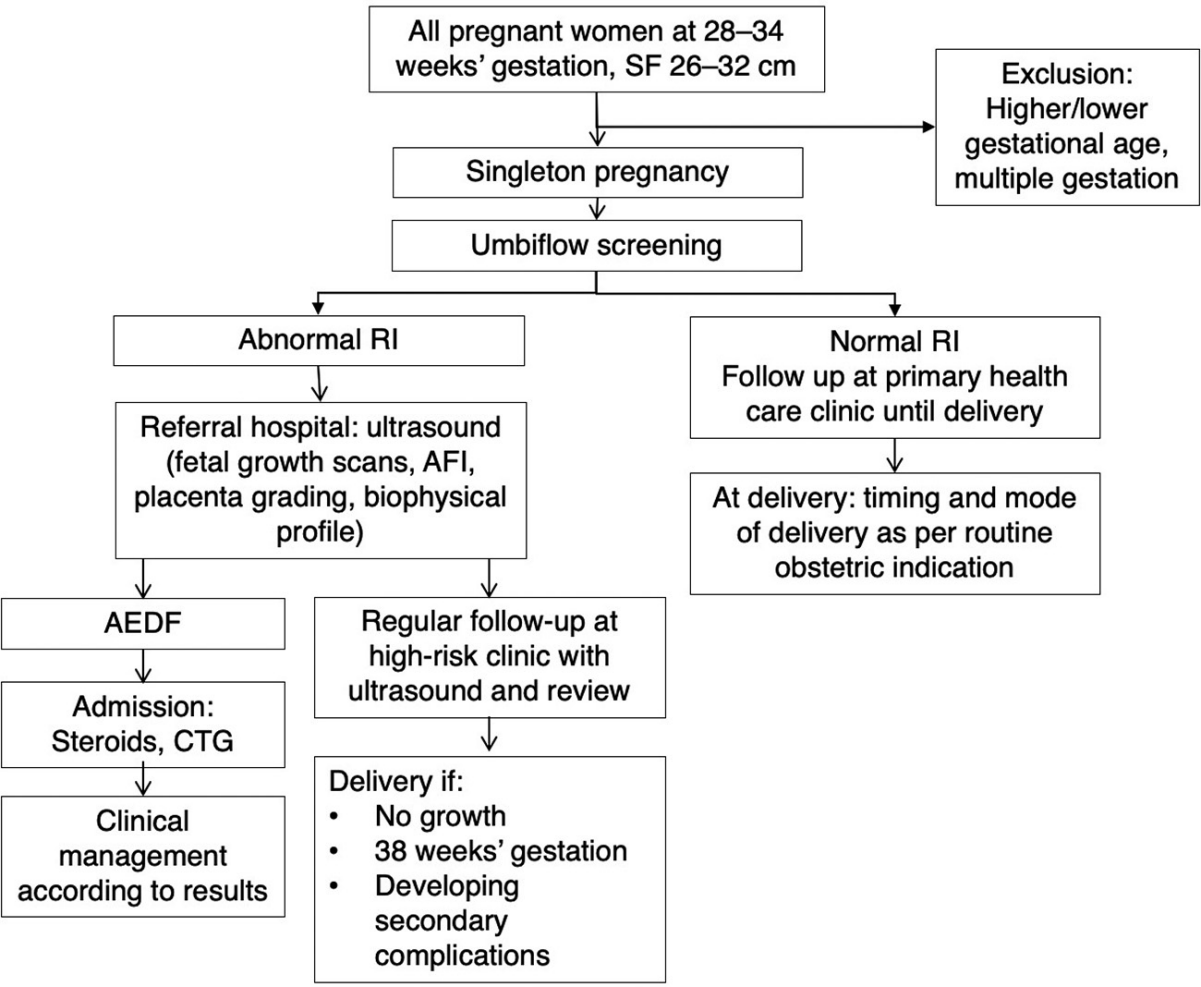
The Umbiflow device, a continuous-wave Doppler ultrasound device used to measure the resistance index in the umbilical artery. © 2022 Council for Scientific and Industrial Research/South African Medical Research Council

## PRACTICAL USE OF THE SCREENING SYSTEM

The practical use of the Umbiflow device is similar to using a doptone to detect the fetal heartbeat. Training on the use of the apparatus takes about 1 week and includes a theoretical introduction to normal physiology, the Doppler principle, and result interpretation, followed by a 3-day hands-on practice under supervision and certification. Health care workers of all levels, including nurses and midwives, can easily be trained in its use. This is considerably less time and effort than that taken to train health care providers in imaging ultrasound. Quality assurance can be ensured as the wave forms are all recorded electronically for later quality control purposes. The apparatus does not need real-time electricity to run as it has a rechargeable battery. Decision making is very simple with a traffic light system with red and amber meaning referral, green being normal. As the system has no imaging ultrasound, it cannot be used to determine the sex of a fetus, which is crucial in settings where sex determination is illegal.

Although screening women with low-risk pregnancies using Doppler ultrasound was initially tested in HICs, its use was later rejected^26^^,^^27^ because it did not adequately detect small-for-gestational age (SGA) babies.^26^ We now understand that SGA is not synonymous with FGR (as discussed later). The studies were also too small and the incidence of abnormal umbilical artery resistance indices or AEDF was too low to detect changes in perinatal mortality.^27^ When the incidence increased, as was the case with high-risk pregnancies, perinatal mortality decreased significantly by 29%.^22^ However, it was never properly tested in LMICs, which carry the largest burden of stillbirths. Until recently, the prevalence of AEDF and abnormal RIs had not been determined (Table).^28^

- Nkosi et al. used CWDU in a sample of 2,868 LRMs in a township in South Africa (an LMIC setting) undergoing a single CWDU screening between 28 and 32 weeks of gestation and found a prevalence of 1.5% for AEDF and 11.7% for abnormal UmA-RI.^21^
- Hlongwane et al. screened 7,088 LRMs with CWDU between 28 and 34 weeks of gestation in 9 different sites across 8 provinces in South Africa^29^ and found a similarly high prevalence of abnormal UmA-RI (13%) and AEDF (1.2%).
- The Umbiflow International study, sponsored by the World Health Organization, conducted in 5 LMICs (Ghana, India, Kenya, Rwanda, and South Africa) reported an overall prevalence of 6.9% of abnormal UmA-RI among LRMs, with the highest prevalence recorded in Ghana.^30^

**TABLE.** Results of Recent Umbiflow Studies

	No. of Participants	Prevalence Abnormal RI	Prevalence AEDF	Mortality Reduction
Mamelodi Nkosi et al.^21^	2,868	11.7%	1.5%	PNMR: −42%
Umbi 9 Hlongwane et al.^29^	7,088	13.0%	1.2%	SBR: −43%
Umbiflow International Vannevel et al.^30^	7,151	6.9%	0.2%	---

Abbreviations: AEDF, absent end diastolic flow; PNMR, perinatal mortality rate; RI, resistance index; SBR, stillbirth rate.

A low-risk pregnancy was defined in all these studies as a pregnant woman attending non-specialist antenatal care clinics and classified as “low risk” at the time of recruitment according to local clinical guidelines, based on their obstetric and clinical assessment. For South Africa, this is guided by the basic antenatal care plus (called “BANC-Plus”) program, following the World Health Organization's recommendations for a positive pregnancy experience.^31^^,^^32^

For the initial 3 studies, normal curves developed in a tertiary hospital were used and the 75th centile was used as the cutoff for abnormality as it was best associated with perinatal mortality; however, the population of women included during the development of the curves was at high risk.^23^^,^^33^

In all the studies, women with an abnormal UmA-RI were referred to a high-risk clinic for review by a doctor (specialist, medical officer, or clinician caring for pregnant women) and further management (Figure).^21^^,^^29^ The screening system must have a referral system and management protocol built in so that the fetus with an abnormal UmA-RI can be referred for further full assessment, which includes a full imaging ultrasound, including fetal weight estimation, pulsed-wave Doppler, and amniotic fluid and placental assessment to examine for FGR. Only with the full system in place can the screening prevent stillbirths.

**FIGURE:**
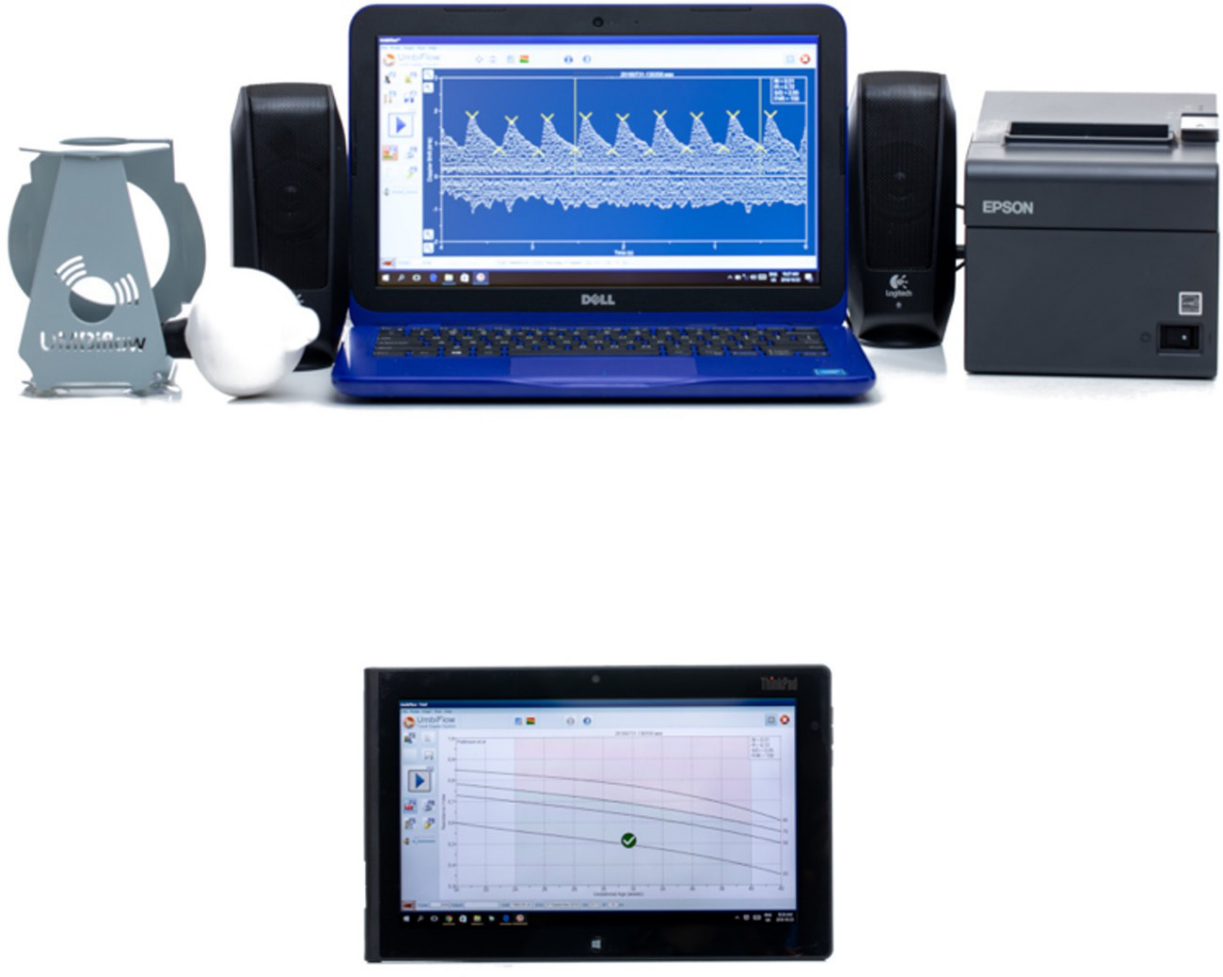
Protocol for Managing Women With an Abnormal Continuous-Wave Doppler Ultrasound Result Abbreviations: AEDF, absent end diastolic flow; AFI, amniotic fluid index; CTG, cardiotocograph; RI, resistance index; SF, symphysis fundal height. Note: Ward-based health outreach teams assisted to trace patients who could not be contacted by other means (except in Umbi-9 study).

### FGR as a Major Cause of Antenatal Stillbirths

A fetus who does not reach its full genetic growth potential is defined as having FGR.^13^ This definition implies that a growth-restricted fetus is not necessarily small but still at risk of adverse outcomes. The fact that a growth-restricted fetus is not always small explains why current techniques such as symphysis-fundal height measurements or measuring fetal size on conventional ultrasound have failed to reduce perinatal mortality. Placental insufficiency is the most common cause of FGR and occurs when the placenta is damaged to such an extent that it cannot provide the fetus with sufficient nutrients and oxygen, ultimately leading to starvation.^34^ As the placental insufficiency progresses, the fetus initially loses fat and glycogen stores, detected by a reduced liver size, followed by a reduction in muscle mass and later in bone mass, with ultimately death (stillbirth) as the most severe consequence. As previously mentioned, AEDF is a measure of end-stage placental disease and, left unmanaged, usually results in a stillbirth. If the prevalence of AEDF is high in a low-risk pregnant population, then FGR is an important cause of stillbirth.

If the prevalence of AEDF is high in a low-risk pregnant population, then fetal growth restriction is an important cause of stillbirth.

When one extrapolates the South African AEDF prevalence to all births in the country per year and assumes, conservatively, that half of the 1 million births annually are from low-risk pregnancies and 1% have undetected AEDF and assumes, conservatively, that half of these fetuses die, then 2,500 stillbirths per year [(500,000 × 0.01) × 0.5)] could be ascribed to AEDF.^29^ Unexplained stillbirths account for approximately 25% of the 20,000 stillbirths annually in South Africa (approximately 5,000 stillbirths),^10^ therefore, approximately 50% (2,500) of the unexplained stillbirths in South Africa could be due to undetected AEDF and could be prevented by CWDU screening.

### Postnatal Diagnosis of FGR

The Umbiflow International study found that fetuses with abnormal UmA-RI were significantly smaller at birth than their normal UmA-RI counterparts, across all birth weight centiles and after correction for sex and gestational age at birth, leading the research team to the conclusion that CWDU has the potential to diagnose FGR before birth.^30^ After birth, FGR due to placental insufficiency can be assessed by studying the infant's body composition, in particular, the fat-free mass (FFM), which is a measure of muscle and bone mass. Unfortunately, this measure is not routinely available, but body composition can be measured in infants by air displacement plethysmography, dual-energy X-ray absorptiometry, or deuterium dilution. A follow-up study of infants of LRMs who were screened with CWDU at 28–34 weeks' gestation reported a significant association between an abnormal UmA-RI in pregnancy and lower infant FFM at 6 weeks, 10 weeks, 14 weeks, and 6 months, pointing toward a starved fetus.^35^ Furthermore, more than 75% of these infants were classified as appropriate-for-gestational age at birth. Interestingly, those infants who were classified at birth as being SGA (see below) but who had a normal UmA-RI during pregnancy had a higher FFM than those infants who were classified as appropriate-for-gestational age with an abnormal UmA-RI, although the numbers were small. This illustrates that screening with CWDU has the ability to detect fetuses with FGR irrespective of their size and birth weight, across all birth weight-for-gestational age centiles.

### Difference Between SGA Babies and FGR

An SGA baby is defined as a baby whose birth weight is less than the 10th centile for its gestational age. Using this definition, 10% of normal babies will classify as SGA. This definition does not satisfy the requirement of a growth-restricted fetus, as, for example, a fetus who is genetically destined to have a birth weight on the 75th centile but who is born on the 25th centile and is not reaching his/her growth potential does not fulfill the criteria of being SGA. This growth-restricted fetus has a much greater risk of adverse outcomes than a fetus who is born on the 8th centile, is SGA, and is not growth-restricted (constitutionally small). The advantage of Doppler ultrasound of the umbilical artery is that it tests placental function rather than fetal size and can detect poor placental function irrespective of the fetal growth centile. This gives screening with Doppler ultrasound a big advantage over screening by measuring size that requires serial measurements to determine the growth pattern, which is much more expensive and difficult for a health system to manage. Thus, the Delphi consensus on defining FGR includes Doppler abnormalities in their definition.^36^ Furthermore, the dropping of centiles (even though not less 10th centile) is included in their definition as contributory parameters in diagnosing FGR supporting the definition of a fetus not reaching its genetic growth potential.

### Impact of CWDU Screening on Low-Risk Pregnancies

Having determined that CWDU screening is potentially worthwhile in geographic locations that have a high prevalence of FGR, the next step was to assess if the knowledge of an abnormal UmA-RI within the clinical care setting actually reduced the stillbirth rate. A cohort analytic study of women attending primary health care antenatal clinics in 9 different geographical areas in diverse settings in South Africa compared a group of LRMs who were between 28 and 34 weeks of gestation and were screened with CWDU to a group of LRMs who were not screened.

Among the CWDU group of 7,088 women, with pregnancy outcomes recorded in 6,536 (92.2%), 66 women had stillbirths, resulting in a stillbirth rate (SBR) of 10.1/1,000 births. Among the control group of 10,832 women, the SBR was 17.8/1,000 births. Hence, screening LRMs with CWDU followed by referral and further management according to the study protocol (Figure) resulted in a decrease in the SBR of 43% (risk ratio [RR]: 0.57, 95% confidence interval [CI]=0.29, 0.85). If all women in the control group who developed subsequent antenatal complications were excluded, there were 9,811 women and 152 stillbirths, giving an SBR of 15.5/1,000 births, which was 35% higher than the CWDU-screened group (RR: 0.65, 95% CI=0.36, 0.94). The cesarean delivery rate was similar between the groups, but there was an increase in the number of low birth weight infants in the CWDU-screened group. However, despite the increase in the use of neonatal services, the neonatal mortality rate did not increase, thus the fetuses who were at risk of stillbirth were not transferred to neonatal deaths (T Hlongwane, unpublished data, 2022).

Screening LRMs with CWDU followed by referral and further management resulted in a decrease in the stillbirth rate of 43%.

Nkosi et al. reported similar results where 2,868 women had CWDU screening and the pregnancy outcomes were available for 2,539 fetuses (88.5%).^21^ There were 29 perinatal deaths in the CWDU group. The perinatal mortality rate for 12,168 women attending primary health care antenatal clinics and their referral hospitals who did not have a CWDU screening was 21.3/1,000 births, significantly higher than in the CWDU group (11.4/1,000 births, RR: 0.58, 95% CI=0.42, 0.81).

Thus, UmA-RI screening using CWDU is an effective tool that detects FGR, and, when the information is used, it prevents stillbirths.

### Additional Effects

There are several known maternal and infant factors that result in FGR, but just as many unknowns; hence, it is currently not feasible to prevent FGR from occurring primarily. However, early detection of FGR and appropriate management of pregnancies enable close fetal monitoring and prevention of stillbirths. If the fetuses survive, these infants can then be further monitored and interventions put in place to optimize postpartum growth and development since it is known that infants who were growth-restricted in utero are at greater risk of stunting, obesity, and cardiovascular diseases (e.g., stroke and hypertension) later in life.^37^^–^^39^ A study done on infants from low-risk pregnancies who had FGR reported that these infants had reduced cognitive ability at 12 years.^40^

Since the “first 1,000 days” are known to be a critical period for growth and development, a simple and effective screening and intervention during this period could play a major role in terms of improved health outcomes, not only in the growing fetus but further into infancy, childhood, and adulthood.

### Potential Negative Effects

The immediate effect of implementing the screening system will be the increased pressure on the local antenatal and neonatal services.

- There will be approximately 10% more referrals of women classified as having low-risk pregnancies to high-risk antenatal clinics for evaluation. This will increase the clinicians' time at the receiving facility. In the previous studies done, a separate clinic was set up for women with an abnormal UmA-RI. Less than 1 of 10 referred women will have a false positive screening test and be referred back to the primary antenatal clinic.
- Furthermore, there will also be more premature and/or growth-restricted neonates requiring clinical management, increasing the use of neonatal services. However, this can be interpreted as positive, as without the screening a number of these neonates would have been stillbirths.

The immediate effect of implementing the CWDU screening system will be the increased pressure on the local antenatal and neonatal services.

The major concerns are: (1) Are the fetuses that are potential stillbirths being transferred to neonatal deaths? and (2) Are there unnecessary interventions that increase maternal and neonatal morbidity? Hlongwane et al. and Nkosi et al. reported no increases in neonatal mortality when the CWDU-screened group was compared to the unscreened group—neither was the cesarean delivery rate significantly different.^21^ Further, Alfirevic et al. did not find a difference in interventions in HICs when those women with normal pregnancies who had a screening Doppler ultrasound were compared to those who did not.^27^ However, as with any new intervention, this aspect will have to be carefully monitored.

Another potential negative effect is that women referred for more intensive evaluation will be more anxious about their pregnancy. Any fetus with an abnormal UmA-RI probably has some degree of FGR, and that infant's FFM will be reduced.^35^ This infant might then be at risk of developing childhood malnutrition or obesity and the woman, knowing this, can then apply appropriate infant feeding practices.

## CONCLUSION

The introduction of a CWDU screening system (screening and follow-up management) for a low-risk pregnant population in an LMIC setting has resulted in a step-change reduction in stillbirths. The screening system is imminently suitable for scaling up in LMICs, and potentially also in HICs. This is achieved by using CWDU as a functional test to detect placental insufficiency that leads to FGR, which is a major cause of unexplained stillbirths.

However, the introduction of the screening system has other effects, namely increasing the need for antenatal referrals of women previously classified as having low-risk pregnancies, increasing the burden on neonatal services, and identifying infants at risk of suboptimal growth and development, together with long-term effects as adults such as obesity, diabetes, hypertension, and stroke. This opens the door to developing interventions to prevent these adverse outcomes, both during pregnancy and after birth. The introduction of CWDU screening is ideally placed to help integrate maternal, fetal, and neonatal services into a seamless “First 1,000 days” care package. Once identified, the at-risk fetuses can be classified as high risk indicating the need for intensified follow-up antenatally, intrapartum, neonatally, and in infancy.

Future research is geared toward identifying the most appropriate health system models within which the CWDU screening system can occur and how best to implement this. Finally, a randomized trial in various LMIC settings needs to be conducted to assess the true role of CWDU screening in preventing stillbirths.

## Notes

**Peer Reviewed**
